# PEDCan: acceptability and feasibility of a mobile application to support primary health-care workers in recognising clinical features of childhood cancer in Uganda

**DOI:** 10.3332/ecancer.2026.2156

**Published:** 2026-06-26

**Authors:** Barnabas Atwiine, Nura Izath, James Ssemitego, Naome Nsiimenta, Wilson Birungi, Anitah Kusaasira, Nuriat Nambogo, Martin Mukama, Santorino Data

**Affiliations:** 1 Department of Paediatrics and Child Health, Mbarara University of Science and Technology, PO Box 1410, Mbarara, Uganda; 2 Department of Paediatrics and Child Health, Mbarara Regional Referral Hospital, PO Box, 40, Mbarara, Uganda; 3 Consortium for Affordable Medical Technologies Uganda (CAMTech Uganda), Mbarara University of Science and Technology, PO Box, 1410, Mbarara, Uganda; 4 Ishongorero Health Centre IV, Mbarara District Local Government, PO Box, 1, Mbarara, Uganda Mbarara, Uganda; 5 Department of Obstetrics and Gynecology, Mulago National Referral Hospital, PO Box 7051, Kampala, Uganda; 6 Center for Innovation and Technology Transfer (CITT), Mbarara University of Science and Technology, PO Box 1410, Mbarara, Uganda

**Keywords:** early diagnosis, timely referral, childhood cancer, primary healthcare workers, Uganda, low- and middle-income countries

## Abstract

**Introduction:**

Early diagnosis of childhood cancer depends on a high index of suspicion and timely referral by primary healthcare workers (PHCWs) to tertiary centers. This study aimed to assess the acceptability and feasibility of a mobile application among PHCWs in recognising cancer-related clinical features in children.

**Methods:**

We developed a mobile application called (PEDCan) to support PHCWs in recognising cancer-related clinical features in children, initiating remote consultations with specialists and referring suspected cases to cancer centers when necessary. In May 2021, we conducted a cross-sectional descriptive study among consenting PHCWs in southwestern Uganda to collect data on their knowledge of childhood cancer, smartphone ownership and internet usage. After training participants on the use of the application through mock clinical vignettes, we assessed their ease of use and readiness to adopt the application. Descriptive statistics were used to calculate the proportions of participants able and willing to use the application. Ethical approval was obtained from the Research and Ethics Committee of Mbarara University of Science and Technology.

**Results:**

A total of 44 PHCWs participated in the study; 25 (56.8%) were nurses or midwives, 8 (18.2%) were clinical officers, 3 (6.8%) were medical doctors and 8 (18.2%) belonged to other professional cadres. All participants had previously heard about childhood cancer, and 28 (63.6%) had previously encountered a child suspected to have cancer. Thirty-eight (86.4%) participants owned a smartphone and used the internet, while 12 (31.6%) reported having constant internet access. Thirty-eight (86.3%) participants found the PEDCan application very easy or easy to use, and all participants expressed willingness to use it.

**Conclusion:**

PHCWs in the participating health facilities were able and willing to use the PEDCan mobile application to recognise childhood cancer clinical features and initiate remote expert consultations using mock vignettes. A pilot study involving real patients would further demonstrate the application’s effectiveness in real-world clinical settings.

## Introduction

Early diagnosis contributes significantly to improved outcomes in children with cancer, as those diagnosed at less advanced stages have better treatment outcomes [3, 4]. For this to happen, primary healthcare workers (PHCWs) must promptly recognise children presenting with clinical features suggestive of cancer and refer to cancer specialists for definitive diagnosis [4, 5]

Globally, an estimated 400,000 children develop cancer each year [6] Of these, approximately 85% live in low- and middle-income countries (LMICs), where access to diagnostic and treatment services remains limited. Consequently, only about half of all children with cancer worldwide are diagnosed, with the majority of missed diagnoses occurring in LMICs [7]

In Uganda, approximately 3,000 children develop cancer annually; however, only about 1,000 (≈30%) are diagnosed [8], most of whom present with advanced disease [9]. This contributes to poor treatment outcomes [10, 11]

In many LMICs, PHCWs have limited or no training in paediatric oncology [12]. As a result, children with cancer are often misdiagnosed and treated for more common conditions or referred through complex and often bureaucratic national referral systems [12–14] Since childhood cancers frequently present with non-specific acute or sub-acute clinical features, they rapidly progress to severe disease or death if not diagnosed and treated promptly [15]. It is therefore critical to equip PHCWs with the knowledge and tools needed to recognise clinical features of cancer and to facilitate direct and timely referral to specialised cancer treatment centers.

Programs in countries such as Botswana [13], South Africa [16] and Rwanda [12] have demonstrated that training PHCWs can improve early recognition and referral of suspected childhood cancer cases. However, these programs have generally not provided ongoing decision-support tools or platforms for remote consultation, leading to attrition of knowledge and skills over time [16]

Telemedicine and mobile health (mHealth) applications have increasingly been used in LMICs to support paediatric care, training and service delivery, with promising results [1]. These technologies can provide decision support and facilitate communication with specialists, thereby helping to reduce disparities in access to specialised care [17–19]

A previous study has explored telehealth-based expert consultation models for childhood cancer [20]. Building on this work, we developed a mobile-based application, called (PEDCan), designed to support PHCWs in identifying clinical features suggestive of childhood cancer, initiating remote consultations with specialists and facilitating appropriate referrals.

We then conducted a study to evaluate the feasibility and acceptability of PEDCan among PHCWs in Southwestern Uganda, to inform the design and implementation of mHealth technologies to improve early diagnosis and treatment outcomes for children with cancer in Uganda and similar settings.

## Methods

### Study setting

This study was conducted in May 2021 in southwestern Uganda, a region with an estimated population of approximately 6 million people [21]. Paediatric oncology services in this region are provided by the Paediatric Cancer Unit (PCU) at Mbarara Regional Referral Hospital (MRRH), located in Mbarara City, about 260 km from the capital, Kampala, and which is one of the three designated comprehensive childhood cancer treatment centers in Uganda and the only one in western Uganda.

Uganda’s healthcare referral system is hierarchical, requiring patients to be referred sequentially from lower-level facilities to higher-level centers when diagnosis or management is not possible at the initial point of care. Three public Level IV Health Centres (HC IVs), Kinoni, Bwizibwera and Mbarara City, were purposively selected for this study because they are located within the catchment area of the PCU at MRRH. Each HC is led by a medical officer and staffed by a multidisciplinary team comprising doctors, clinical officers, nurses/midwives and laboratory personnel, with an average of 35 PHCWs. These facilities provide inpatient and outpatient services, including general wards, operating theatres, outpatient clinics and pharmacies and manage common childhood and maternal conditions. They receive referrals from lower-level health facilities and private clinics, as well as patients presenting directly from their homes. Cases requiring specialised care are referred to district or regional referral hospitals.

### Study participants

This was a cross-sectional study assessing the feasibility and acceptability of the PEDCan mobile application. All PHCWs at the three selected HC IVs were eligible to participate and were approached during their routine weekly continuous professional development (CPD) meetings. At each facility, the first 15 PHCWs to arrive were consecutively recruited and provided written informed consent. Participants initially completed the first part of a structured questionnaire capturing baseline knowledge of childhood cancer, prior experience managing children with suspected cancer and their access to and use of smartphones and the internet.

### Training and intervention

Following recruitment, all CPD attendees received a didactic lecture on the recognition of childhood cancer delivered by a paediatric oncologist. After the lecture, only the enrolled participants remained and underwent hands-on training in the use of the PEDCan mobile application.

Participants were then provided with mock clinical vignettes, which included children with and without clinical features suggestive of cancer. Participants were asked to put the information from these cases into the PEDCan application.

### Data collection, management and analysis

Subsequently, the participants were interviewed using the second part of the questionnaire (adapted from a previous study that evaluated the use of another mobile application at our institution) to assess their experience using the application, perceived usability and their willingness to adopt the tool in routine clinical practice. Data from completed questionnaires were entered into Microsoft Excel spreadsheets and analysed using descriptive statistics. Frequencies and proportions were calculated for responses to key variables related to feasibility and acceptability.

### Ethical considerations

Ethical approval was obtained from the Research and Ethics Committee (REC) of Mbarara University of Science and Technology (MUST). Administrative permission was also obtained from the management of the three participating health facilities before study initiation.

### Description of the PEDCan mobile application

**Figure 1.**
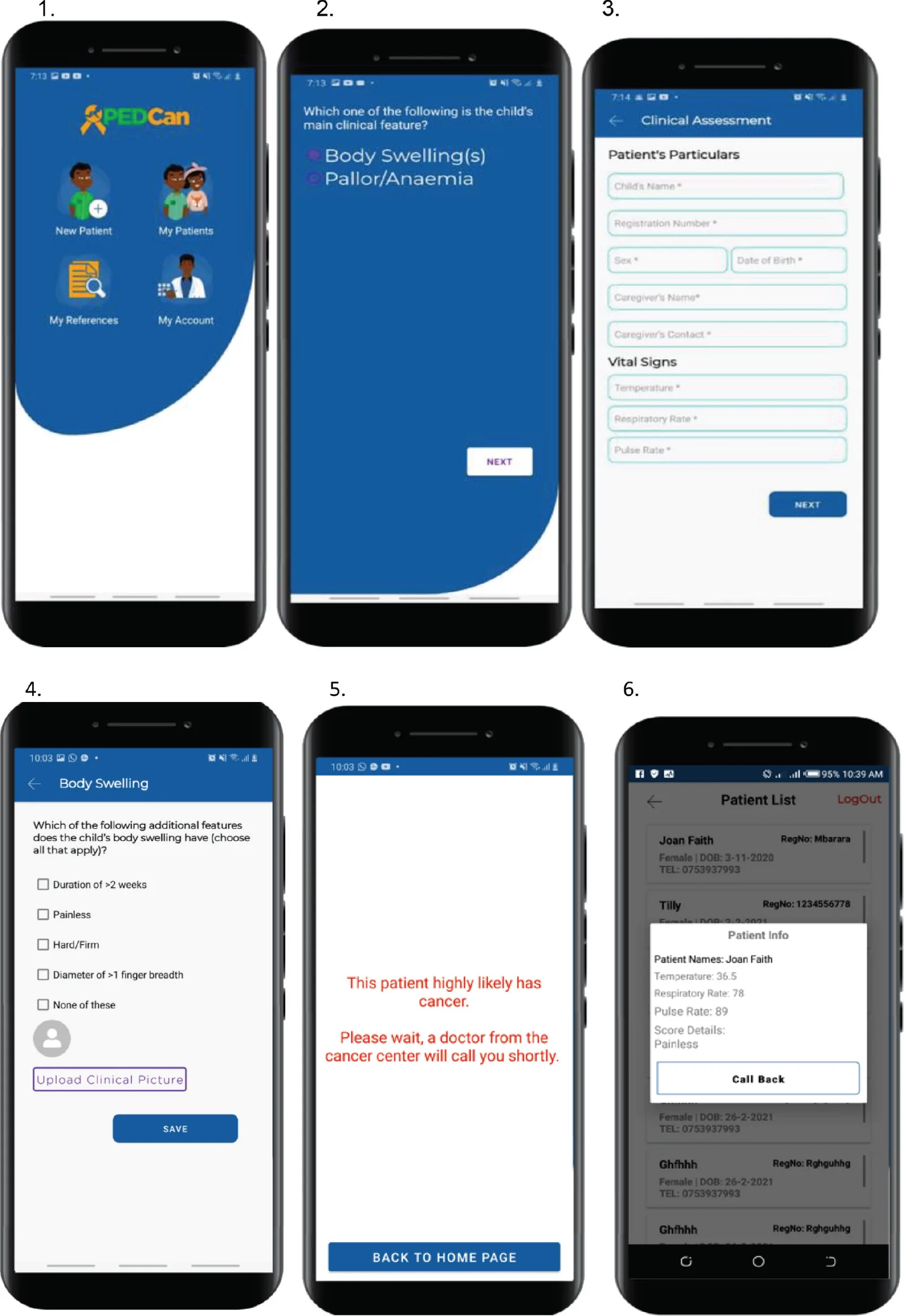
Interfaces of the PEDCan application. 1. Home screen with the PEDCan main features (New Patient, My Patient, My References and My Account). 2. Screen for clinical features. 3. Screen for clinical assessment. 4. Screen for further assessments. 5. Feedback screen. 6. Specialist’s interface with patient list

As shown in Figure 1, PEDCan application users Users register on the platform as either PHCWs or cancer specialists. Upon login, PHCWs can access: *New Patient*, *My Patients*, *My References* and *My Account;* while Specialists can access: *Patient List*

The application is based on the clinical premise that childhood cancers commonly present with either body masses/lumps (i.e., solid tumours and lymphomas) or pallor (i.e., leukaemias) [15]

After a PHCW has assessed a child with either a mass/lump or pallor, they select the child’s most prominent feature of the two (either a mass or pallor) within the application. Each category includes additional criteria suggestive of malignancy: mass-related clinical features, which include duration >2 weeks, diameter of >2 cm and being painless, firm/hard consistency or pallor-related features, which include fever >2 weeks in duration, hepatosplenomegaly/lymphadenopathy, bleeding tendencies and limb pains.

Based on combinations of these features, the application classifies patients into three categories: unlikely, likely or highly Likely to have cancer. For patients classified as *Likely* or *Highly Likely*, the application transmits clinical information to the nearest cancer specialist. The specialist receives an alert via a pre-recorded voice notification and initiates a phone call to the PHCW for remote consultation and guidance on referral, if necessary. For patients classified as *Unlikely*, the application generates a prompt advising consideration of alternative diagnoses. All data entered into the application is securely stored on an encrypted Linux-based server.

### Expected impact of PEDCan

PEDCan is designed to facilitate direct communication between PHCWs and cancer specialists, improve the timeliness and appropriateness of referrals, promote earlier diagnosis and initiation of treatment, support continuous learning through embedded reference materials and enhance retention of knowledge and clinical skills among PHCWs.

## Results

A total of 45 PHCWs consented to participate in the study, and all, except one, underwent the training, completed both questionnaires and their data were analysed.

### Demographic characteristics of the study participants

The demographic characteristics of the 44 participants are shown in Table 1. Their median (range) age was 33 (22–55) years. Twenty-eight (63.4%) were females, 25 (56.8%) nurses/midwives, 8 (18.2%) clinical officers, 3 (6.8%) doctors, 4 (9%) social workers, 2 (4.5%) laboratory assistants, 1 (2.2%) pharmacy assistant and 1 (2.2%) anesthetic assistant.

### Awareness and experiences of participants regarding childhood cancer

The responses of the participants regarding their awareness and experiences with childhood cancer are shown in Table 2. All (100%) of the participants had previously heard about cancer, and 39 (88.6%) knew that children suffer from it. Thirty-two (72.7%) participants knew that childhood cancer presents with body masses; 21 (47.7%) with weight loss; 20 (45.5%) with anemia; 17 (38.6%) with pain; 13 (29.5%) with fever; 6 (13.6%) with general body weakness; 5 (11.4%) with bleeding tendencies; 5 (11.4%) with breathing difficulties and 4 (9%) with headaches.

Twenty-eight (63.6%) participants had previously seen a child who was suspected of having cancer, with 21 (75%) saying that such children had body masses, 10 (35.7%) pain, 8 (28.6%) anemia and 3 (10.7%) breathing difficulties.

### Smart phone ownership and internet usage

As shown in Table 3, 38 (86.4%) participants owned a smartphone at the time of the interview and used the internet. Of the 38 participants, 12 (31.6%) accessed the internet all the time, 17 (44.7%) only during the day or night and 9 (23.9%) less frequently. Twenty-six (68.4%) had previously used another health-related mobile application on their phones.

### Ease of use and acceptance of the PEDCan mobile application

Twenty-four (54.5%), 14 (31.8%) and 6 (13.6%) participants found the learning of the use of PEDCan very easy, easy and somewhat easy, respectively. Forty-one (93.1%) participants said they would recommend its use in primary health care facilities.

All (100%) of the participants, after undergoing training in the use of the PEDCan application, reported that they thought that the application would help them detect cancer-related clinical features in children because it has a guide of the symptoms and signs, it was easy to use and it creates direct consultation with a specialist. They all said that the application would change the way they manage children suspected of having cancer, and were willing to and interested in using it.

## Discussion

This study demonstrated a high level of ease with which PHCWs were able to learn and operate the PEDCan mobile application. The findings suggest that PEDCan is a simple and user-friendly tool for PHCWs, who are often the first and sometimes the only health workers to encounter sick children at primary health care facilities. When equipped with appropriate training and access to smartphones and the internet, PHCWs can use PEDCan to obtain timely specialist input, facilitating rapid clinical decision-making. Similar benefits of mHealth tools have been reported in childhood cancer care [19] and other broader disease management contexts [15, 22–25].

Importantly, PEDCan enables direct communication between PHCWs and cancer specialists, thereby bypassing the traditional stepwise referral system. This has the potential to reduce delays associated with sequential referrals and promote earlier access to definitive care at specialised cancer treatment centers.

Our study also demonstrated universal acceptability of the PEDCan application among PHCWs. Participants highlighted several reasons for this high level of acceptance, including the integration of cancer-related signs and symptoms within the application, its ease of use and its ability to facilitate direct consultation with specialists. These findings are consistent with previous studies showing that mobile applications can enhance PHCWs’ confidence, support remote consultations and improve patient outcomes [1, 2]. The ONCOpeds application, for example, has similarly been shown to be highly acceptable and feasible for facilitating remote consultations between PHCWs and paediatric oncology specialists [20].

Diagnosing childhood cancer in LMICs remains challenging, as most of its clinical features mimic common paediatric illnesses [15], increasing the likelihood of misdiagnosis or delayed diagnosis. Our findings suggest that PEDCan, when combined with targeted training in recognising childhood cancer features, can serve as an effective telemedicine intervention to reduce missed and delayed diagnoses. Additionally, the application enhances communication between PHCWs and specialists, strengthening the overall referral pathway.

Another important advantage of PEDCan is its inclusion of simplified reference materials, which PHCWs can repeatedly access during routine practice. This feature may support ongoing learning and improve long-term retention of knowledge and clinical skills, reducing the need for frequent refresher training sessions.

One potential limitation to the widespread implementation of PEDCan is limited internet connectivity in rural areas. However, this barrier is gradually diminishing. Currently, approximately 50% of Uganda’s geographic area is covered by 3G networks, and internet penetration, estimated at 26.2%, is increasing rapidly (by approximately 14% annually) [25]. Notably, the majority of participants in this study (86.4%) owned smartphones and used the internet, suggesting a strong foundation for scaling such interventions.

Additionally, the sample size was relatively small and limited to three HC located within the catchment area of the PCU at MRRH. These facilities may have better access to internet infrastructure compared to more remote settings, potentially limiting generalisability. Future studies should include more geographically diverse and rural populations, where smartphone ownership and internet connectivity may be lower.

A key strength of this study is that participants were recruited directly from their clinical work settings, ensuring that the findings reflect real-world usability. Furthermore, participants demonstrated adequate baseline competence in smartphone use, which supports the feasibility of implementing PEDCan in similar contexts.

## Conclusion and recommendations

The PEDCan mobile application is highly acceptable and feasible for use among PHCWs in southwestern Uganda. The study demonstrated strong enthusiasm and readiness among participants to adopt the application in routine clinical practice.

These findings support the need for a larger-scale pilot study involving more PHCWs across a wider geographic area to evaluate the effectiveness of PEDCan in real-world clinical settings. Such a study would provide critical evidence on its impact on early diagnosis, referral patterns and treatment outcomes for children with cancer. Ultimately, PEDCan has the potential to contribute significantly to improving the detection and management of childhood cancer in Uganda and other low-resource settings.

## List of abbreviations

*CAMTech,* Consortium for Affordable Medical Technologies in Uganda; *CPD, Continuous professional development; HCs, Health centres; LMIC, Low-and-middle income countries; MRRH, Mbarara Regional Referral Hospital; MUST, Mbarara University of Science and Technology; PCU, Paediatric cancer unit; PEDCan, Paediatric cancer; PHCWs, Primary health care workers; REC, Review and Ethics Committee; UCI, Uganda Cancer Institute.*

## Conflicts of interest

The authors declare no conflicts of interest.

## Funding

The development of the PEDCan mobile application and the study were supported by the First Mile Program of Massachusetts General Hospital (MGH), in Boston, USA, through the Consortium of Affordable Medical Technologies in Uganda (CAMTech Uganda) of Mbarara University of Science and Technology.

## Figures and Tables

**Table 1.** Demographic characteristics of the study participants.

Characteristic	Frequency (n)	Percentage (%)
**Age: Median (range)**	33(22–55) years	
**Sex, Female**	28	68.2
**Cadre**		
Nurse/midwife	25	56.8
Clinical officers	8	18.2
Doctors	3	6.8
Social workers	4	9.0
Laboratory assistant	1	2.2
Pharmacy assistant	1	2.2
Anesthetic assistant	1	2.2

**Table 2.** Participants’ awareness about and previous experiences with childhood cancer.

Attribute	n (%)
A. Awareness and experiences of participants regarding childhood cancer	
Have you ever heard about cancer?	
Yes	44 (100)
Do you know if children suffer from cancer?	
Yes	39 (88.6)
No	3 (6.8)
Not sure	2 (4.5)
Which symptoms and signs do children with cancer present with?	
Body masses	32 (72.7)
Weight loss	21 (47.7)
Anaemia	20 (45.5)
Pain	17 (38.6)
Fever	13 (29.5)
Body weakness	6 (13.6)
Bleeding tendencies	5 (11.4)
Breathing difficulties	5 (11.4)
Headache	4 (9)
Have you ever seen a child suspected to have cancer?	
Yes	28 (63.6)
How did the child above present with? (*n*-28)	
Body masses	21 (75)
Pain	10 (35.7)
Anemia	8 (28.6)
Breathing difficulties	3 (10.7)
B. Smartphone ownership and internet usage	
Do you currently own a smart phone?	
Yes	38 (86.4)
If you have a smart phone, how often do you use internet with it? (*n* = 38)	
All the time	12 (31.6)
Only during the day or night	17 (44.7)
Less than once a day	9 (23.7)
Have you ever used a health-related mobile application on your phone? (*n*-38)	
Yes	26 (68.4)

**Table 3.** Participants’ ease of and willingness to use the PEDCan application.

Attribute	n (%)
A. Ease of use of PEDCan mobile application	
Will PEDCan help you identify children suspected with cancer?	
Yes	44 (100)
Why do think PEDCan will help you to identity children suspected with cancer?	
It has a guide to symptoms and signs of cancer	22 (50)
It is easy to use	20 (45.5)
Directly connects to a specialist	17 (38.6)
Will PEDCan change your management of children suspected with cancer?	
Yes	44 (100)
How easy did you find learning how to use PEDCan?	
Very easy	24 (54.5)
Easy	14 (31.8)
Somewhat easy	6 (13.6)
B. Willingness and interest to use PEDCan mobile application	
Are you willing and interested in using PEDCan?	
Yes	44 (100)
Do you recommend PEDCan to be used in primary health facilities?	
Yes	41 (93.2)
No	2 (4.5)
Not sure	1 (2.3)
